# Vaccination Coverage by Age 24 Months Among Children Born in 2019 and 2020 — National Immunization Survey-Child, United States, 2020–2022

**DOI:** 10.15585/mmwr.mm7244a3

**Published:** 2023-11-03

**Authors:** Holly A. Hill, David Yankey, Laurie D. Elam-Evans, Michael Chen, James A. Singleton

**Affiliations:** 1 Immunization Services Division, National Center for Immunization and Respiratory Diseases, CDC.

**Summary**

**What is already known about this topic?**

The Advisory Committee on Immunization Practices recommends vaccines against 15 potentially serious diseases by the age of 24 months.

**What is added by this report?**

Estimated coverage with most childhood vaccines was similar among children born during 2019–2020 compared with those born during 2017–2018, with only a few exceptions. Disparities in coverage by race and ethnicity, poverty status, insurance status, and urbanicity persist, with a widening of the gap among some subgroups evident over time.

**What are the implications for public health practice?**

Universal and equitable access to vaccination will require overcoming economic, logistic, and attitudinal obstacles to ensure that all children are protected from vaccine-preventable diseases.

## Abstract

National Immunization Survey-Child data collected in 2022 were combined with data from previous years to assemble birth cohorts and assess coverage with routine vaccines by age 24 months by birth cohort. Overall, vaccination coverage was similar among children born during 2019–2020 compared with children born during 2017–2018, except that coverage with both the birth dose of hepatitis B vaccine and ≥1 dose of hepatitis A vaccine increased. Coverage was generally higher among non-Hispanic White (White) children (2–21 percentage points higher than coverage for non-Hispanic Black or African American, Hispanic or Latino, and non-Hispanic American Indian/Alaska Native [AI/AN] children), children living at or above poverty (3.5–22 percentage points higher than coverage for children living below the federal poverty level), privately insured children (2.4–38 percentage points higher than coverage for children with Medicaid, other insurance, or no insurance), and children in urban areas (3–16.5 percentage points higher than coverage for children living in rural areas). Coverage with the full series of *Haemophilus influenzae* type b conjugate vaccine was lower among AI/AN children compared with White children. Trends in vaccination coverage disparities across categories of race and ethnicity, health insurance status, poverty status, and urbanicity were evaluated for the 2016–2020 birth cohorts. Fewer than 5% of 168 trends examined were statistically significant, including six increases (widening of the coverage gap) and one decrease (narrowing of the gap). Analyses revealed a widening of the gap between children living at or above the poverty level (higher coverage) and those living below poverty (lower coverage), for several vaccines. Socioeconomic, demographic, and geographic disparities in vaccination coverage persist; addressing them is important to ensure protection for all children against vaccine-preventable disease.

## Introduction

The World Health Organization describes immunization as a “global health and development success story,” responsible for preventing 3.5–5 million deaths each year.* In the United States, the Advisory Committee on Immunization Practices (ACIP) recommends vaccines against 15 potentially serious diseases by age 24 months^†^ (*1*). For nearly 30 years, the National Immunization Survey-Child (NIS-Child) has monitored coverage with ACIP-recommended childhood vaccines in the United States. National coverage estimates provide an overall picture of the strength of the U.S. immunization program and insight into coverage with new vaccines. Stratification by sociodemographic and geographic variables allows for identification of subpopulations at higher risk for disease because of lower vaccination coverage. NIS-Child data have been used previously to assess the impact of the COVID-19 pandemic on coverage with childhood vaccinations (*2*). This assessment did not identify any consistent or persistent decline in vaccination coverage associated with the COVID-19 pandemic at the national level. Among certain subgroups, however, coverage was lower during the pandemic period. For example, coverage with the combined seven-vaccine series by age 24 months decreased 4–5 percentage points among children living below the federal poverty level or in rural areas.

## Methods

### Data Collection

NIS-Child uses random-digit-dialing to identify U.S. households that contain children aged 19–35 months.^§^ A telephone survey^¶^ is conducted with the parent or guardian who is most knowledgeable about the child’s immunization history, and consent is requested to contact the child’s vaccine providers. If consent is granted, a questionnaire is mailed to all the child’s providers to obtain vaccination information, which is synthesized to create the child’s comprehensive vaccination history. Children born during 2019–2020 were identified using data collected during 2020–2022. The household interview response rate** for 2022 was 25.1%, and 49.7% of children with completed parent or guardian interviews had adequate provider data,^††^ resulting in data from 27,733 children available for analysis.

### Data Analysis

All NIS-Child coverage estimates are based on information supplied by providers. Kaplan-Meier techniques were used to estimate vaccination coverage by age 24 months, except for the birth dose of hepatitis B vaccine (HepB)^§§^ and rotavirus vaccine.^¶¶^ Because of a change in ACIP recommendations and an extremely long period of eligibility for catch-up vaccination, coverage with ≥2 doses of hepatitis A vaccine (HepA) was estimated by age 35 months (the maximum age available) as well as by age 24 months.*** The significance of coverage differences was assessed using z-tests; p<0.05 was considered statistically significant. Vaccination coverage among children born during 2019–2020 was compared with that among children born during 2017–2018. Five-year trends in coverage and in socioeconomic and demographic disparities by year of birth were evaluated by fitting a linear regression model and testing for the significance of the slope (average annual percentage point change [AAPPC]). Analyses used weighted data and were performed using SAS software (version 9.4; SAS Institute) and SUDAAN software (version 11; RTI International). This activity was reviewed by CDC, deemed not research, and was conducted consistent with applicable federal law and CDC policy.^†††^

## Results

### Children Born During 2019–2020

**National vaccination coverage.** Estimated coverage with most childhood vaccines was similar among children born during 2019–2020 and those born during 2017–2018, with the exception of a 3.3 percentage point increase in coverage with the HepB birth dose and a 1.5 percentage point increase in coverage with ≥1 dose of HepA (Table 1). The proportion of children completely unvaccinated by age 24 months remained at 1%. Coverage among children born during 2019–2020 exceeded 90% for ≥3 doses of poliovirus vaccine (93.0%), ≥3 doses of HepB (92.1%), ≥1 dose of measles, mumps, and rubella vaccine (MMR) (91.6%), and ≥1 dose of varicella vaccine (VAR) (91.1%). The lowest coverage estimates were observed for ≥2 doses of influenza vaccine (61.3%) and for the combined seven-vaccine series^§§§^ (69.1%).

**TABLE 1:** Estimated vaccination coverage by age 24 months,* among children born during 2017–2018 and 2019–2020 for selected vaccines and doses — National Immunization Survey-Child, United States, 2018–2022

Vaccine/Dose	% (95% CI)		
	Birth years^†^		Difference
	2017–2018	2019–2020	(2017–2018 to 2019–2020)
**DTaP^§^**			
≥3 doses	93.6 (93.1 to 94.1)	93.8 (93.1 to 94.4)	0.2 (−0.6 to 1.0)
≥4 doses	81.6 (80.8 to 82.4)	81.0 (79.9 to 82.0)	−0.6 (−2.0 to 0.7)
**Poliovirus (≥3 doses)**	92.6 (92.0 to 93.2)	93.0 (92.3 to 93.6)	0.4 (−0.5 to 1.2)
**MMR (≥1 dose)^¶^**	91.3 (90.7 to 91.9)	91.6 (90.8 to 92.2)	0.2 (−0.7 to 1.2)
Hib**			
Primary series	92.8 (92.2 to 93.4)	93.4 (92.7 to 94.0)	0.5 (−0.3 to 1.4)
Full series	79.6 (78.7 to 80.5)	79.1 (78.0 to 80.1)	−0.6 (−2.0 to 0.8)
**HepB**			
Birth dose^††^	78.1 (77.2 to 79.0)	81.5 (80.5 to 82.4)	3.3 (2.0 to 4.6)^§§^
≥3 doses	91.8 (91.2 to 92.4)	92.1 (91.4 to 92.7)	0.3 (−0.6 to 1.2)
**VAR (≥1 dose)^¶^**	90.5 (89.9 to 91.2)	91.1 (90.4 to 91.8)	0.6 (−0.4 to 1.6)
**PCV**			
≥3 doses	92.4 (91.8 to 93.0)	92.8 (92.1 to 93.5)	0.4 (−0.5 to 1.3)
≥4 doses	82.2 (81.4 to 83.1)	82.7 (81.7 to 83.7)	0.5 (−0.9 to 1.8)
**HepA**			
≥1 dose	86.9 (86.2 to 87.7)	88.4 (87.6 to 89.2)	1.5 (0.4 to 2.5)^§§^
≥2 doses^¶¶^	46.4 (45.4 to 47.5)	47.7 (46.4 to 48.9)	1.3 (−0.4 to 2.9)
≥2 doses (by age 35 mos)^¶¶^	78.1 (76.9 to 79.3)	80.0 (78.4 to 81.6)	1.9 (−0.1 to 3.9)
**Rotavirus** (by age 8 mos)***	75.7 (74.8 to 76.6)	76.6 (75.6 to 77.7)	0.9 (−0.4 to 2.3)
**Influenza (≥2 doses)^†††^**	60.6 (59.6 to 61.6)	61.3 (60.1 to 62.5)	0.7 (−0.9 to 2.2)
**Combined seven-vaccine series^§§§^**	70.0 (69.0 to 71.0)	69.1 (67.9 to 70.2)	−0.9 (−2.5 to 0.6)
**No vaccinations^¶¶¶^**	1.0 (0.9 to 1.2)	1.0 (0.8 to 1.2)	0.0 (−0.3 to 0.2)

**Abbreviations:** DTaP = diphtheria and tetanus toxoids and acellular pertussis vaccine; HepA = hepatitis A vaccine; HepB = hepatitis B vaccine; Hib = *Haemophilus influenzae* type b conjugate vaccine; MMR = measles, mumps, and rubella vaccine; PCV = pneumococcal conjugate vaccine; VAR = varicella vaccine.

* Includes vaccinations received by age 24 months, except for the HepB birth dose, rotavirus vaccination, and ≥2 HepA doses by age 35 months. For all vaccines except the HepB birth dose and rotavirus vaccination, the Kaplan-Meier method was used to estimate vaccination coverage to account for children whose vaccination history was ascertained before age 24 months (35 months for ≥2 HepA doses).

^†^ Data for the 2017 birth year are from survey years 2018, 2019, and 2020; data for the 2018 birth year are from survey years 2019, 2020, and 2021; data for 2019 birth year are from survey years 2020, 2021, and 2022; data for the 2020 birth year are considered preliminary and are from survey years 2021 and 2022 (data from survey year 2023 are not yet available).

^§^ Includes children who might have been vaccinated with diphtheria and tetanus toxoids vaccine or diphtheria, tetanus toxoids, and pertussis vaccine. Healthy People 2030 target for ≥4 doses of DTaP by age 2 years is 90%. https://health.gov/healthypeople/objectives-and-data/browse-objectives/vaccination

^¶^ Includes children who might have been vaccinated with MMR and varicella combination vaccine. Healthy People 2030 target for ≥1 dose of MMR by age 2 years is 90.8%. https://health.gov/healthypeople/objectives-and-data/browse-objectives/vaccination

** Hib primary series: receipt of ≥2 or ≥3 doses, depending on product type received; full series: primary series and booster dose, which includes receipt of ≥3 or ≥4 doses, depending on product type received.

^††^ One dose HepB administered from birth through age 3 days.

^§§^ Statistically significantly different (p<0.05) from zero.

^¶¶^ Before 2020, the first Hep A dose was recommended at age 12–23 months, with the second dose given 6–18 months after the first, depending upon the product type received. In 2020, recommendation revised to 2 doses between ages 12 and 23 months, ≥6 months apart. Because children in this analysis were vaccinated under both recommendations, coverage estimates for both 24 months and 35 months are provided.

*** Includes ≥2 doses of Rotarix monovalent rotavirus vaccine or ≥3 doses of RotaTeq pentavalent rotavirus vaccine; if any dose in the series is either RotaTeq or unknown, the default is to a 3-dose series. The maximum age for the final rotavirus dose is 8 months, 0 days.

^†††^ Influenza vaccine doses must be ≥24 days apart (4 weeks with a 4-day grace period); doses could have been received during two influenza seasons.

^§§§^ The combined seven-vaccine series (4:3:1:3*:3:1:4) includes ≥4 doses of DTaP, ≥3 doses of poliovirus vaccine, ≥1 dose of measles-containing vaccine, the full series of Hib (≥3 or ≥4 doses, depending on product type), ≥3 doses of HepB, ≥1 dose of VAR, and ≥4 doses of PCV.

^¶¶¶^ Healthy People 2030 target for children who get no recommended vaccines by age 2 years is ≤1.3%. https://health.gov/healthypeople/objectives-and-data/browse-objectives/vaccination

**Vaccination coverage by selected sociodemographic characteristics and geographic locations.** Among children born during 2019–2020, coverage was higher among those who were privately insured compared with uninsured children and children insured by Medicaid or other insurance^¶¶¶^ for all vaccines except the HepB birth dose, which did not differ between privately insured children and those who were insured by Medicaid (Table 2). Compared with children with private insurance (0.6% unvaccinated), a higher proportion of uninsured children (6.0%) and children on Medicaid (1.2%) received no vaccinations by age 24 months.

**TABLE 2:** Estimated vaccination coverage by age 24 months* among children born during 2019–2020,^†^ by selected vaccines and doses and health insurance status^§^ — National Immunization Survey-Child, United States, 2020–2022

Vaccine/Dose	Health insurance status, % (95% CI)			
	Private only (Ref) n = 15,668	Any Medicaid n = 9,682	Other insurance n = 1,961	Uninsured n = 422
**DTaP^¶^**				
≥3 doses	96.3 (95.7–96.9)	92.2 (91.1–93.2)**	92.1 (89.5–94.3)**	80.4 (72.7–87.1)**
≥4 doses	87.3 (86.1–88.4)	76.6 (74.8–78.3)**	76.3 (72.3–80.1)**	61.3 (52.3–70.4)**
**Poliovirus (≥3 doses)**	95.6 (94.9–96.2)	91.3 (90.1–92.3)**	91.6 (88.9–93.8)**	80.0 (72.2–86.9)**
**MMR (≥1 dose)^††^**	94.6 (93.9–95.3)	89.6 (88.4–90.7)**	88.9 (85.7–91.6)**	78.3 (70.1–85.6)**
**Hib^§§^**				
Primary series	95.7 (95.0–96.4)	91.9 (90.9–92.9)**	91.8 (89.3–94.0)**	78.8 (71.0–85.8)**
Full series	84.4 (83.2–85.6)	75.1 (73.3–76.9)**	76.7 (72.9–80.3)**	61.9 (53.1–70.8)**
**HepB**				
Birth dose^¶¶^	83.0 (81.8–84.2)	81.6 (80.1–83.0)	74.9 (70.8–78.5)**	63.7 (53.7–72.7)**
≥3 doses	93.7 (92.9–94.5)	91.3 (90.2–92.3)**	90.8 (88.2–93.1)**	76.2 (68.1–83.6)**
**VAR (≥1 dose)^††^**	94.0 (93.2–94.8)	89.5 (88.2–90.6)**	87.7 (84.4–90.5)**	76.5 (68.5–83.8)**
**PCV**				
≥3 doses	95.6 (94.8–96.3)	91.0 (89.8–92.1)**	91.3 (88.6–93.5)**	79.9 (72.0–86.8)**
≥4 doses	89.3 (88.3–90.4)	78.1 (76.3–79.8)**	79.3 (75.7–82.8)**	55.3 (46.2–64.8)**
**HepA**				
≥1 dose	91.2 (90.3–92.1)	86.7 (85.3–87.9)**	86.0 (82.8–88.9)**	72.3 (63.5–80.5)**
≥2 doses***	51.9 (50.3–53.5)	44.7 (42.7–46.7)**	43.9 (39.6–48.4)**	—^†††^
≥2 doses (by age 35 mos)***	85.4 (83.7–87.0)	76.3 (73.5–78.9)**	75.4 (69.7–80.7)**	—^†††^
**Rotavirus** (by age 8 mos)^§§§^	84.1 (82.9–85.3)	71.2 (69.5–72.9)**	72.9 (68.5–76.9)**	52.0 (42.6–61.2)**
**Influenza (≥2 doses)^¶¶¶^**	75.5 (74.1–76.9)	49.2 (47.3–51.2)**	61.4 (57.1–65.6)**	37.8 (29.4–47.6)**
Combined seven-vaccine series****	76.6 (75.1–78.0)	63.6 (61.6–65.5)**	66.2 (62.0–70.4)**	42.5 (33.9–52.3)**
**No vaccinations**	0.6 (0.5–0.8)	1.2 (0.8–1.5)**	0.8 (0.5–1.2)	6.0 (3.4–9.5)**

**Abbreviations:** DTaP = diphtheria and tetanus toxoids and acellular pertussis vaccine; HepA = hepatitis A vaccine; HepB = hepatitis B vaccine; Hib = *Haemophilus influenzae* type b conjugate vaccine; MMR = measles, mumps, and rubella vaccine; PCV = pneumococcal conjugate vaccine; Ref = referent group; VAR = varicella vaccine.

* Includes vaccinations received by age 24 months, except for the HepB birth dose, rotavirus vaccination, and ≥2 HepA doses by age 35 months. For all vaccines except the HepB birth dose and rotavirus vaccination, the Kaplan-Meier method was used to estimate vaccination coverage to account for children whose vaccination history was ascertained before age 24 months (35 months for ≥2 HepA doses).

^†^ Data for the 2019 birth year are from survey years 2020, 2021, and 2022; data for the 2020 birth year are considered preliminary and are from survey years 2021 and 2022 (data from survey year 2023 are not yet available).

^§^ Children’s health insurance status was reported by parent or guardian. “Other insurance” includes the Children’s Health Insurance Program, military insurance, coverage through the Indian Health Service, and any other type of health insurance not mentioned elsewhere.

^¶^ Includes children who might have been vaccinated with diphtheria and tetanus toxoids vaccine or diphtheria, tetanus toxoids, and pertussis vaccine.

** Statistically significant (p<0.05) difference compared with the Ref.

^††^ Includes children who might have been vaccinated with MMR and VAR combination vaccine.

^§§^ Hib primary series: receipt of ≥2 or ≥3 doses, depending on product type received; full series: primary series and booster dose, which includes receipt of ≥3 or ≥4 doses, depending on product type received.

^¶¶^ One dose HepB administered from birth through age 3 days.

*** Before 2020, the first Hep A dose was recommended at age 12–23 months, with the second dose given 6–18 months after the first, depending upon the product type received. In 2020, recommendation was revised to 2 doses between ages 12 and 23 months, ≥6 months apart. Because children in this analysis were vaccinated under both recommendations, coverage estimates for both 24 months and 35 months are provided.

^†††^ Estimate was not available because the unweighted sample size for the denominator was <30, 95% CI half width divided by the estimate was >0.588, or 95% CI half-width was ≥10.

^§§§^ Includes ≥2 doses of Rotarix monovalent rotavirus vaccine or ≥3 doses of RotaTeq pentavalent rotavirus vaccine; if any dose in the series is either RotaTeq or unknown, the default is to a 3-dose series. The maximum age for the final rotavirus dose is 8 months, 0 days.

^¶¶¶^ Influenza vaccine doses must be ≥24 days apart (4 weeks with a 4-day grace period); doses could have been received during two influenza seasons.

**** The combined seven-vaccine series (4:3:1:3*:3:1:4) includes ≥4 doses of DTaP, ≥3 doses of poliovirus vaccine, ≥1 dose of measles-containing vaccine, the full series of Hib (≥3 or ≥4 doses, depending on product type), ≥3 doses of HepB, ≥1 dose of VAR, and ≥4 doses of PCV.

Numerous disparities in coverage by race and ethnicity were observed. Most notably, non-Hispanic Black or African American (Black) children, Hispanic or Latino, and non-Hispanic American Indian or Alaska Native (AI/AN) children all had lower coverage with ≥4 doses of diphtheria and tetanus toxoids and acellular pertussis vaccine (DTaP), ≥4 doses of pneumococcal conjugate vaccine (PCV), rotavirus vaccine, ≥2 doses of influenza vaccine, and the combined seven-vaccine series compared with non-Hispanic White (White) children. Coverage with the full series of *Haemophilus influenzae* type b conjugate vaccine (Hib) was lower by 12.1 percentage points among AI/AN children compared with White children. (Supplementary Table 1, https://stacks.cdc.gov/view/cdc/134544). Children living below the federal poverty level had lower coverage than children living at or above the poverty level for all vaccines except the HepB birth dose. Compared with children living in a metropolitan statistical area (MSA)**** principal city, those residing in a non-MSA had lower coverage with approximately one half of the vaccines monitored by NIS-Child. Wide variation in coverage estimates was also observed by jurisdiction (Supplementary Table 2, https://stacks.cdc.gov/view/cdc/134545), especially for ≥2 doses of influenza vaccine, which ranged from 33.0% (Mississippi) to 85.9% (Connecticut).

### Trends by Birth Cohort

Coverage by birth cohort during 2011–2020 was stable for a majority of vaccines, although a decrease of 5.1 percentage points was observed for ≥2 doses of influenza vaccine among children born in 2020 compared with those born in 2019 (Figure). Examination of trends in overall coverage for the five most recent birth cohorts (2016–2020) revealed increases for the HepB birth dose (1.7 percentage points per year), ≥1 dose of HepA (0.9 percentage points per year), and ≥2 doses of HepA (0.8 percentage points per year); no decreases were found (Supplementary Table 1, https://stacks.cdc.gov/view/cdc/134544).

**FIGURE:**
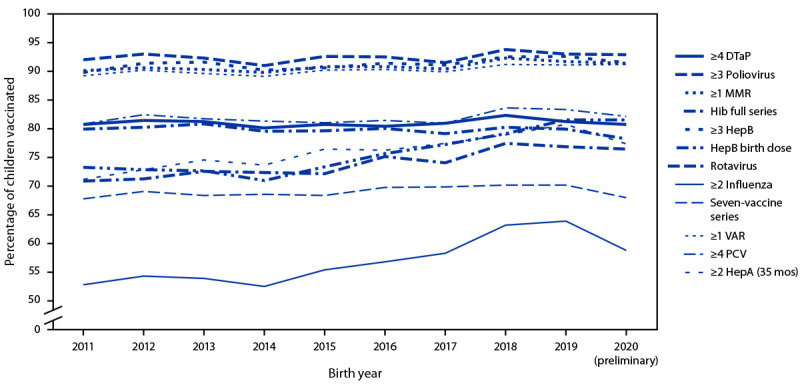
Estimated coverage with selected individual vaccines*^,^^†^^,^^§^^,^^¶^^,^^**^^,^^††^^,^^§§^ and a combined vaccine series^¶¶^ by age 24 months, by birth year*** — National Immunization Survey-Child, United States, 2012–2022 **Abbreviations:** DTaP = diphtheria and tetanus toxoids and acellular pertussis vaccine; HepA = hepatitis A vaccine; HepB = hepatitis B vaccine; Hib = *Haemophilus influenzae* type b conjugate vaccine; MMR = measles, mumps, and rubella vaccine; PCV = pneumococcal conjugate vaccine; VAR = varicella vaccine. * Includes vaccinations received by age 24 months, except for the HepB birth dose, rotavirus vaccination, and ≥2 HepA doses by 35 months. For all vaccines except the HepB birth dose and rotavirus vaccination, the Kaplan-Meier method was used to estimate vaccination coverage to account for children whose vaccination history was ascertained before age 24 months (35 months for ≥2 HepA doses). ^†^ Includes children who might have been vaccinated with diphtheria and tetanus toxoids vaccine or diphtheria, tetanus toxoids, and pertussis vaccine. ^§^ Includes children who might have been vaccinated with MMR and varicella combination vaccine. ^¶^ Hib full series: primary series and booster dose, which includes receipt of ≥3 or ≥4 doses, depending on product type received. ** One dose HepB administered from birth through age 3 days. ^††^ Includes ≥2 doses of Rotarix monovalent rotavirus vaccine or ≥3 doses of RotaTeq pentavalent rotavirus vaccine; if any dose in the series is either RotaTeq or unknown, the default is to a 3-dose series. The maximum age for the final rotavirus dose is 8 months, 0 days. ^§§^ Influenza vaccine doses must be ≥24 days apart (4 weeks with a 4-day grace period); doses could have been received during two influenza seasons. ^¶¶^ The combined seven-vaccine series (4:3:1:3*:3:1:4) includes ≥4 doses of DTaP, ≥3 doses of poliovirus vaccine, ≥1 dose of measles-containing vaccine, the full series of Hib (≥3 or ≥4 doses, depending on product type), ≥3 doses of HepB, ≥1 dose of VAR, and ≥4 doses of PCV. *** Children born in 2011 are included in survey years 2012, 2013, and 2014; children born in 2012 are included in survey years 2013, 2014, and 2015; children born in 2013 are included in survey years 2014, 2015, and 2016, children born in 2014 are included in survey years 2015, 2016, and 2017; children born in 2015 are included in survey years 2016, 2017, and 2018; children born in 2016 are included in survey years 2017, 2018, and 2019; children born in 2017 are included in survey years 2018, 2019 and 2020; children born in 2018 are included in survey years 2019 and 2020, and 2021; children born in 2019 are included in survey years 2020, 2021, and 2022; data for children born in 2020 are considered preliminary and are from survey years 2021 and 2022 (data from survey year 2023 are not yet available).

Coverage was also estimated by the five most recent birth cohorts within each category of the sociodemographic variables (race and ethnicity, poverty level, health insurance status, and MSA status) (Supplementary Table 1, https://stacks.cdc.gov/view/cdc/134544). Positive linear trends were observed for the HepB birth dose for multiple subgroups of children, including non-Hispanic White and multiple race children, children living at or above the poverty level, privately insured and Medicaid-insured children, and those living in an MSA principal city or an MSA nonprincipal city. Increased coverage with ≥1 dose of HepA (White, any Medicaid insurance, and MSA nonprincipal city), ≥2 doses of HepA (White, at or above poverty level, private insurance only, and non-MSA), and rotavirus vaccine (Black) was observed over time. No decreases were seen for any of the combinations of vaccines and categories of sociodemographic variables.

In addition, trends in disparities were assessed for 2016–2020 birth cohorts (Supplementary Table 3, https://stacks.cdc.gov/view/cdc/134546). Among 168 trends evaluated, six increases (widening of the coverage gap between a variable category and the referent group) and one decrease (narrowing of the gap) were identified.^††††^ The most common of these was the disparity in coverage by poverty status, with a widening of the gap in coverage with ≥2 HepA doses, ≥2 influenza vaccine doses, and the combined seven-vaccine series between children living below poverty and those living at or above poverty.

## Discussion

This report incorporates NIS-Child data collected in 2022 to assess vaccination coverage, disparities in vaccination coverage, and 5-year trends in coverage and disparities in coverage among children born during 2016–2020. For most recommended childhood vaccines, coverage has remained high and stable for a number of years. Among children born during 2019–2020, coverage exceeded 70% for all vaccines except ≥2 doses of influenza vaccine (61.3%) and the combined seven-vaccine series (69.1%). HepB birth dose coverage has been trending upward for several years, exceeding 80% for the first time in 2019. Coverage with ≥1 dose of HepA has increased more slowly, but if the current trend continues, coverage will exceed 90% among children born in 2022. Among children born during 2019–2020, Healthy People 2030^§§§§^ objectives have been met for coverage with ≥1 dose of MMR by age 24 months (≥90.8%) and for the proportion of children who receive no recommended vaccines by age 24 months (≤1.3%), but not for coverage with ≥4 DTaP doses (≥90.0%).

Disparities persist in vaccination coverage by race and ethnicity, poverty status, MSA status, and health insurance status and are often substantial. Lower coverage with the full series of Hib among AI/AN children compared with White children is particularly concerning given the sharply elevated incidence of Hib disease in the AI/AN population.^¶¶¶¶^ The largest observed coverage disparities were for ≥2 doses of influenza; influenza vaccination coverage varied widely by jurisdiction as well, with a range of 52.9 percentage points across the United States. Analysis of 5-year trends revealed that only a small proportion of the disparities involving sociodemographic variables changed over time, although it appears that children living below the poverty level might be losing ground compared with children with higher family incomes. Disparities such as these have been documented previously (*3*,*4*). Concern over financial barriers to vaccination led to the creation of the Vaccines for Children (VFC) program,***** which covers the cost of recommended vaccines for eligible children. The program appeared successful in reducing racial and ethnic disparities in coverage (*5*), but additional efforts will be needed to close the remaining coverage gaps. CDC is currently working with partners, such as state Medicaid programs, the Indian Health Service, and the Association of Immunization Managers, to increase awareness of the VFC program (*6*).

Universal and equitable access to vaccination will require overcoming often interrelated economic, logistical, and attitudinal obstacles. Interviews with parents identified issues such as appointment scheduling challenges, incomplete knowledge of the schedule of recommended vaccines, limited availability and high cost of child care for other children in the household, and lack of transportation as factors that limit access to care (*7*). Strategies that have been found useful in addressing barriers to vaccination include identifying venues other than physician offices for the administration of vaccines (such as health departments, child care centers, and pharmacies), strong provider recommendations, reminder and recall interventions, standing orders, vaccination status review at every health care encounter, and expanded use of immunization information systems to provide consolidated immunization histories (*8*,*9*).

### Limitations

The findings in this report are subject to at least three limitations. First, the low household interview response rate (21%–25% over survey years 2018–2022) and the availability of adequate provider data for only 49%–54% of those who completed interviews during these survey years creates the possibility of selection bias. Second, use of weighting to account for nonresponse and households without telephones might not have completely eliminated bias because of these factors. Finally, coverage estimates could be incorrect if some providers did not return vaccination history questionnaires or if administered vaccines were not documented accurately. Total survey error for the 2022 survey year data was assessed and demonstrated that coverage was underestimated by 1.7 percentage points for ≥1 dose of MMR, 3.3 percentage points for the HepB birth dose, and 9.2 percentage points for the combined seven-vaccine series (*10*). An analysis of change in bias of vaccination coverage estimates from 2021 to 2022 determined that a meaningful change in bias was unlikely.

### Implications for Public Health Practice

Overall coverage with recommended childhood vaccinations remains high; however, persistent disparities in coverage among children in racial and ethnic minority groups, as well as those who are not privately insured, who live in rural areas, and who live below the poverty level must be addressed to ensure that all children are protected from vaccine-preventable diseases. Data from immunization information systems can be used to identify local areas and population subgroups with lower vaccination coverage; children in these groups might be more susceptible to outbreaks of vaccine-preventable diseases.^†††††^ More extensive use of the VFC program, interventions to improve vaccine confidence, enhanced flexibility in scheduling vaccination appointments, and expanded options for the place of vaccination will aid in making the U.S. immunization program more accessible and equitable for all (*7**–**9*).

## Footnotes

* https://www.who.int/health-topics/vaccines-and-immunization#tab=tab_1

† Vaccination against COVID-19 was recommended for children aged 6 months–4 years in June 2022 (https://www.cdc.gov/mmwr/volumes/71/wr/mm7126e2.htm). Because the recommendation was not in effect until midway through the data collection year, an accurate estimate of COVID-19 vaccine coverage cannot be calculated from the 2022 data.

§ Estimates for U.S. Department of Health and Human Services regions, states, selected local areas, and the U.S. territories of Guam and Puerto Rico (https://www.cdc.gov/vaccines/imz-managers/coverage/childvaxview/data-reports/index.html). Certain local areas that receive federal Section 317 immunization funds are sampled separately and included in the NIS-Child sample every year (Chicago, Illinois; New York, New York; Philadelphia County, Pennsylvania; Bexar County, Texas; and Houston, Texas). National estimates in this report exclude U.S. territories.

¶ The NIS-Child used a landline-only sampling frame during 1995–2010. During 2011–2017, the survey was conducted using a dual-frame design, with both mobile and landline sampling frames included. In 2018, the NIS-Child returned to a single-frame design, with all interviews conducted by mobile telephone.

** The Council of American Survey Research Organizations (CASRO) household response rate is calculated as the product of the resolution rate (percentage of the total telephone numbers called that were classified as nonworking, nonresidential, or residential), screening completion rate (percentage of known households that were successfully screened for the presence of age-eligible children), and the interview completion rate (percentage of households with one or more age-eligible children that completed the household survey). For CASRO response rates and the proportions of children with household interviews that had adequate provider data for survey years 2015–2020. https://www.cdc.gov/vaccines/imz-managers/nis/downloads/NIS-PUF20-DUG.pdf

†† Children with at least one vaccination reported by a provider and those who had received no vaccinations were considered to have adequate provider data. “No vaccinations” indicates that the vaccination status is known because the parent or guardian indicated there were no vaccinations and the providers returned no immunization history forms or returned them indicating that no vaccinations had been administered.

§§ Coverage with the birth dose of HepB is measured as the proportion of infants or newborns who received a dose of HepB by age 3 days.

¶¶ Rotavirus is assessed at age 8 months to reflect the maximum age at administration recommended by ACIP.

*** Before 2020, the first dose of HepA was recommended at age 12–23 months, with the second dose administered 6–18 months after the first, depending upon the product type received. During 2020, recommendation was revised to 2 doses between ages 12 and 23 months, ≥6 months apart. Because children in this analysis were vaccinated under both recommendations, coverage estimates for both <24 months and <35 months are provided.

††† 45 C.F.R. part 46.102(l)(2); 21 C.F.R. part 56; 42 U.S.C. Sect. 241(d); 5 U.S.C. Sect. 552a; 44 U.S.C. Sect. 3501 et seq.

§§§ The combined seven-vaccine series (4:3:1:3*:3:1:4) includes ≥4 doses of DTaP vaccine; ≥3 doses of poliovirus vaccine; ≥1 dose of measles-containing vaccine; ≥3 or ≥4 doses (depending upon product type) of Hib; ≥3 doses of HepB; ≥1 dose of VAR; and ≥4 doses of PCV.

¶¶¶ “Other insurance” includes the Children’s Health Insurance Program, military insurance, coverage through the Indian Health Service, and any other type of health insurance not mentioned elsewhere.

**** MSA status was determined based on household reported city and county of residence and was grouped into three categories: MSA principal city, MSA nonprincipal city, and non-MSA. MSAs and principal cities were as defined by the U.S. Census Bureau (https://www.census.gov/programs-surveys/metro-micro.html). Non-MSA areas include urban populations not located within an MSA, as well as completely rural areas.

†††† Significant trends in disparities were associated with a statistically significant AAPPC. Positive trends were seen for the HepB birth dose among children living in an MSA nonprincipal city (AAPPC = 0.9); ≥1 dose of HepA among non-Hispanic multiple race children (AAPPC = 0.7); ≥2 doses of HepA among children living below the poverty level (AAPPC = 2.1); ≥2 doses of influenza vaccine among children living below the poverty level (AAPPC = 1.7) and among children with any Medicaid insurance (AAPPC = 1.6); and the combined seven-vaccine series among children living below the poverty level (AAPPC = 1.0). A negative trend was seen for rotavirus vaccine among children with any Medicaid insurance (AAPPC = −1.3).

§§§§
https://health.gov/healthypeople/objectives-and-data/browse-objectives/vaccination

¶¶¶¶
https://cih.jhu.edu/programs/hibvax-study/

***** Eligible children include those aged ≤18 years who are Medicaid-eligible, uninsured, AI/AN, or insured by health plans that do not fully cover routine immunization (if vaccination is received at a federally qualified health center or a rural health clinic). https://www.cdc.gov/vaccines/programs/vfc/

†††††
https://www.cdc.gov/measles/cases-outbreaks.html
